# Smart Camera Aware Crowd Counting via Multiple Task Fractional Stride Deep Learning †

**DOI:** 10.3390/s19061346

**Published:** 2019-03-18

**Authors:** Minglei Tong, Lyuyuan Fan, Hao Nan, Yan Zhao

**Affiliations:** School of Electronics and Information Engineering, Shanghai University of Electric Power, Shanghai 200090, China; fan_ly@mail.shiep.edu.cn (L.F.); nanhao@mail.shiep.edu.cn (H.N.); yanzhao79@hotmail.com (Y.Z.)

**Keywords:** crowd counting, multiple task learning, smart camera, fractional stride network

## Abstract

Estimating the number of people in highly clustered crowd scenes is an extremely challenging task on account of serious occlusion and non-uniformity distribution in one crowd image. Traditional works on crowd counting take advantage of different CNN like networks to regress crowd density map, and further predict the count. In contrast, we investigate a simple but valid deep learning model that concentrates on accurately predicting the density map and simultaneously training a density level classifier to relax parameters of the network to prevent dangerous stampede with a smart camera. First, a combination of atrous and fractional stride convolutional neural network (CAFN) is proposed to deliver larger receptive fields and reduce the loss of details during down-sampling by using dilated kernels. Second, the expanded architecture is offered to not only precisely regress the density map, but also classify the density level of the crowd in the meantime (MTCAFN, multiple tasks CAFN for both regression and classification). Third, experimental results demonstrated on four datasets (Shanghai Tech A (MAE = 88.1) and B (MAE = 18.8), WorldExpo’10(average MAE = 8.2), NS UCF_CC_50(MAE = 303.2) prove our proposed method can deliver effective performance.

## 1. Introduction

The automatic analysis of crowd has been a particular security technique in the current intelligent surveillance literature [1], which will prevent severe accidents by providing crucial information about the number of people and crowd density in a scene. Therefore, crowd counting and analysis has become an active topic in the computer vision literature due to its extensive application video surveillance, traffic monitoring, public safety, and urban planning [2]. However, the current intelligent surveillance system is still incapable of handling a large-scale crowded environment with severe occlusion and non-uniformity [3].

People-Counting technology can be generalized into two kinds of literature: detection methods and regression methods. Traditional approaches for crowd counting from images relied on hand-crafted representations to extract low-level features. These features were then mapped for counting or generating density maps via various regression techniques. 

Some of the early methods [4] relied on pedestrian detection indirectly, such as HOG (Histograms of oriented gradients) features, can get a more accurate number of people when the crowd is sparse or there are no apparent overlap between people, but the results will be questionable when the group becomes denser. The detection-based model typically employs sliding window-based detection algorithms to count people in an image [5]. In Reference [6], a technique that unites features of HOG and color histogram is presented, which can eliminate a few errors in detection utilizing coalescing SVM (Support Vector Machine) of the two kinds of features. Generally some regression-based methods have also been adopted through regression models such as Gaussian processing regression, linear regression, SVM regression and so forth, to find the function of crowd features and count, which possess relatively simple relationship between the statistical characteristic of pixel [7] and the crowd density, with strong generalization ability in classification. However, the estimation effect would be unfortunate in densely crowded scenes if the foreground was not extracted well since these methods rely on the extraction of the front. These methods are seriously influenced by the existence of high-density crowd and background disturbance. To overcome these obstacles, researchers attempted to count by regression where they learn a mapping to their counts via features extracted from local image patches to their counts [8]. The crowd estimation method based on image texture feature [4,9] can solve the problem of unsatisfactory prediction in the dense crowd to a certain extent, but this method has unfavorable performance in sparse crowd estimation. Besides, it is quite easy to be disturbed by background texture on account of the texture features directly extracted from the original image. Unlike counting by detection, estimating crowd counts without recognizing the location of each person via regression, preparatory works employ edge and texture features such as HOG and LBP to learn the mapping from image patterns to corresponding crowd counts [10,11,12].

To the best of our knowledge, the traditional methods are hampered in dense crowds, and the performance is far from expected. Inspired by the success of Convolutional Neural Networks (CNN) for various computer vision tasks, many types of CNN-based methods have been developed, some new techniques are related to visual understanding [13,14], while some techniques are devoted to overcoming the difficulties of crowd counting [15]. Concerning receptive field and the loss of details issues, the algorithms that can achieve better accuracy still have some limited capabilities, and specific CNN-based methods individually meet the problem of utilizing features at different scales via multi-column or recover spatial resolution via transposed convolutions in CNN-based cascaded multi-task learning (Cascaded-MTL) network [16,17]. Though these methods demonstrated robustness to similar issues, they are still restricted to the various scales and limited capacity to learn well-generalized models. Multi-source information is utilized [18] to regress the crowd counts in high dense crowd images. An end-to-end CNN model adopted from AlexNet [19] is constructed recently for counting in extremely crowded scenes. Later, instead of regressing the count directly, the appearances of crowds are prepared by regressing the CNN feature maps as crowd density maps. Similar frameworks are also developed in Reference [20], where a Hydra-CNN model is designed to compute the crowd in different scenes. Better performance can be obtained by further exploiting switching structures or contextual correlations using LSTM [21,22,23]. Though estimation by regression is reliable in crowded scenes, without information of object location, their predictions for low-density crowds tend to be overestimated. The firmness of such kinds of methods depends upon the stability of statistical benchmarks, while in such scenarios, the instance number is too small to help explore its fundamental analytical philosophy. Detection and regression methods ignore critical spatial information present in the images as they regress on the global count. Hence, Lempitsky et al. [10] proposed a new approach to learning a linear mapping between local patch features and corresponding object density maps to incorporate spatial information present in the images.

Most recently, Sam et al. [22] propose the Switch-CNN using a specific density level classifier to choose different regression model for particular input patches. Sindagi et al. [23] proposed a Contextual Pyramid CNN, which uses CNN networks to regress context at different layers for obtaining lower counting error and better quality density maps. These two solutions produce state-of-the-art performance, and both of them used multi-column based architecture (MCNN) and density level classifier. However, we observe several disadvantages in these approaches: Multi-column CNN’s are hard to train according to the training method described in the work in Reference [16]. Such inflated network structure requires more time to train. Both solutions need density level classifier before sending pictures in the MCNN. However, the granularity of density level is hard to define in real-time full scene analysis since the number of objects keeps varying on a large scale. Also, using a classifier means more columns need to be implemented which makes the design more complicated and causes more overabundance. These works spend a large portion of parameters on density level classification to label the input regions instead of feeding parameters into the final density map generation. Since the branch structure in MCNN is not efficient for generating a density map is unfavorable to the ultimate accuracy. Considering the above drawbacks, we propose a novel approach to concentrate on encoding the broader and deeper features in clustered scenes and generating a high-quality density map. The front-end VGG-16 [24] of the model named CSRNet in Reference [25] outputs a picture, which is 1/8 size of the original input. As discussed in CSRNet, the output size will be further shrunken if proceeding to stack more convolution layers and pooling layers (necessary components in VGG-16), additionally, it is difficult to generate high-quality density maps, so the back-end employs dilated convolutions used to extract more in-depth salient information and improve output resolution.

In this paper, the proposed work is motivated by the idea of multiple task learning and dilated convolution in Cascaded-MTL [18] and CSRNet.

Dilated convolutions make it possible to acquire a larger receptive field, meanwhile applying small convolutions. Yu et al. [26] first attempted to utilize dilated convolutions in semantics segmentation, in which a hybrid dilated convolution (HDC) framework was developed to enlarge the receptive fields effectively. A further discussion on dilated convolution was expounded by Chen et al. [27], who employed atrous convolution in cascade or in parallel to capture multi-scale context by adopting multiple dilated rates to handle the problem of segmenting objects at various scales. These researches have a significant effect on choosing convolution types and the formation of model structures in our paper. Furthermore, the problem that some details of the image would be lost in the down-sampling, which is worth taking into consideration.

Multiple task learning can boost the classification and regression performance of a deep network. Eigen et al. [28] presented a multi-scale CNN for simultaneously predicting depth, surface normal and semantic labels from an image. They apply CNN at three different scales where the output of the smaller scale network is fed as input to the larger one. UberNet [29] employ a similar concept of training low, mid, and high-level vision tasks at the same time. It fuses different scales of the image pyramid for multi-task training on diverse sets. Gkiovani et al. [30] model a CNN for pose estimation and action detection, using features only from the last layer. Ranjan et al. [31] present an algorithm for simultaneous face detection, landmarks localization, pose estimation, and gender recognition using deep convolutional neural networks (CNN). The proposed method fuses the intermediate layers of a deep CNN which boosts up their performances.

However, we address designing an easy-trained CNN-based density map manager in this paper. The dilated convolution layers are deployed as the front-end to enlarge receptive fields and fractional stride convolution layers as the back-end to restore its spatial resolution. By making use of such structure, we significantly reduce parameters which make CAFN trained easily. The proposed MTCAFN extends the CAFN to multiple tasks not only in density map regression but also in density-level classification. Moreover, MTCAFN is developed as an extended network, uniting the shallow features extracted from the shallow sub-net with the broad features obtained from deep sub-net contributes to improving the network effectively via updating parameters. Furthermore, the proposed model uses the multi-task learning method to get the ability of density-level classification; finally, the loss of details will be restored by transposed convolutional layers as much as possible. This new improvement could significantly boost the performance of crowd counting. Besides, we outperform the previous crowd counting solution lower MAE, frequently used benchmark datasets, respectively. To summarize, we make the following contributions:

(1) A principled way of combining different size dilated convolution layers.
(2) Multiple tasks learning framework not only regress the density map, but classify the density level of the crowd.
(3) Some results could outperform state-of-the-art methods on several benchmarking datasets.
(4) The proposed model has been verified in a camera with an embedded system.

The rest of the paper is stated as follows. Section 2 introduces the fabric and configuration of the proposed system and model. Section 3 presents the experimental results on several datasets. In Section 4, we conclude the paper.

## 2. Proposed Framework

The framework proposed in this paper is designed to estimate the crowd density if the number of crowd people exceeds a threshold, defined as representing an abnormality.

### 2.1. Smart Camera System Configuration

**Figure 1:**
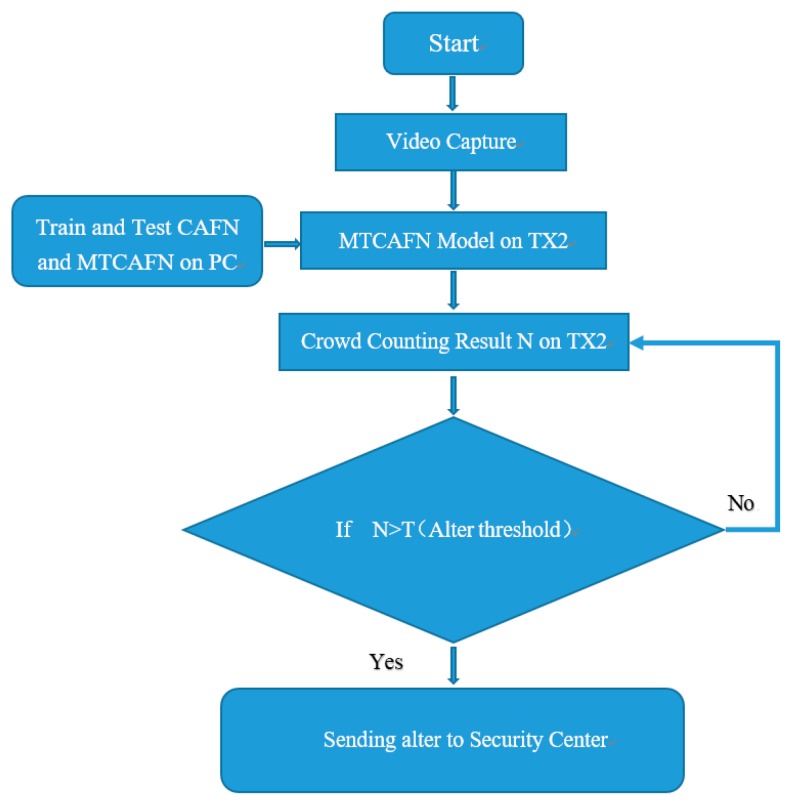
Flowchart of the Edge Computing.

The proposed system could accurately predict the crowd by using smart systems with a single camera and Nvidia Tx2 boards. First, the crowd scene images from three data sets will be inputted into our proposed model CAFN and MTCAFN on PC for training and testing, where we apply 1/10 of training set as the validation set. The trained model is transferred to the Tx2 board for online computing. After that, the real crowd scene will be captured by a camera and the final result will be predicted by our model. When the output counting is larger than the altering threshold T, the system will send the early warning to the surveillance center. The whole flowchart of the alerting system is shown in Figure 1.

### 2.2. CAFN Architecture

In the framework CAFN, the fundamental idea is to deploy a double-column dilated CNN for recording high-level features with larger receptive fields and producing high-quality density maps without obviously expanding network complexity. In this subsection, we firstly introduce the architecture, a network whose input is the image and the output is a density map of the crowd (say how many people per square meter), and then obtain the headcount by integration, then we present the corresponding training method.

Inspired by the idea of CSRNet, we utilized dilated convolutions as the front-end of CAFN because of its greater receptive fields, unlike adopting dilated convolution to capture more features when the resolution has been dropped off to a shallow level in CSRNet. Atrous convolutions are primarily made use of in this paper, which intent to gain more image information from an original image, then the transposed convolutional layer is to enlarge the size of image and up-sampling the previous layer’s output to supplement the loss of details.

In this paper, for attaining the training dataset, we crop 9 patches from each image at different locations with 1/4 size of the inputting images. The first four patches contain four quarters of the image without overlapping while the other five pieces are randomly cropped from the input image. Based on three branches of MCNN, we add dilation rate to filters to enlarge its receptive fields. To reduce net parameters, we consider four types of double-column association as experimental objects, which will be discussed in detail in Section 4. After extracting features from filters with different scales, we try to deploy transposed convolutional layers as the back-end for maintaining the output resolution. We choose a relatively better model, taking into account the stability of the model, of which MAE is not the lowest (but MSE is the lowest) by comparing different groups.

**Figure 2:**
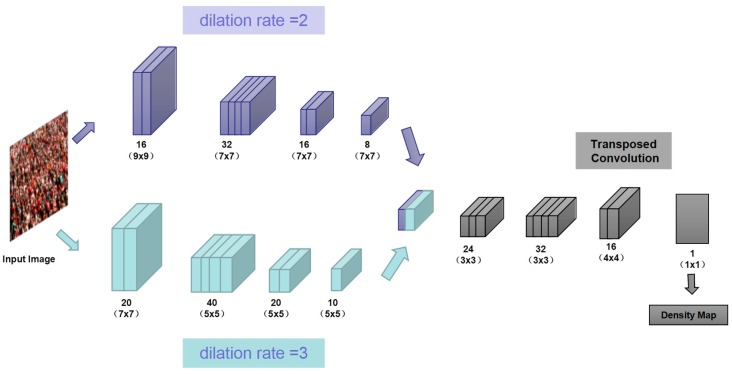
The structure of the proposed double-column convolutional neural network for crowd density map estimation.

The overall structure of our CAFN is illustrated in Figure 2. It contains double parallel columns whose filters are with different dilate rates and local receptive fields of different sizes. Double convolutional columns are merged for fusing features from different scales, here we use the function (torch.cat) to concatenate matrices (feature maps) output from double columns, respectively, on the first dimension. For simplification, we use the same network structures for all columns (i.e., conv–pooling–conv–pooling) except for the sizes and numbers of filters. Max pooling is applied for each 2 × 2 region, and Parametric Rectified linear unit (PReLU) is adopted as the activation function because of its favorable performance for CNN. To reduce the computational complexity (the number of parameters to be optimized), we apply less number of filters for a convolutional layer with larger filters. We stack the output feature maps of all convolutional layers and map them to a density map. To map the feature maps to the density map, we adopt filters whose sizes are 1 × 1. The configuration of our network is shown below in detail (See Table 1).

All convolutional layers use padding to keep the previous size. The convolutional layers’ parameters are denoted as “conv (kernel size) @ (number of filters)”, max-pooling layers are conducted over a 2 × 2 pixel window with stride 2. The fractional stride convolutional layer is denoted as “Conv Transposed (kernel size) @ (number of filters)”, and PReLU is used as a non-linear activation layer.

Then Euclidean distance is exploited to measure the difference between the estimated density map and ground truth. The loss function is defined as follows:

(1) $$L\left(\theta \right)=\frac{1}{2N}{\sum_{i=1}^{N}\left\|Y\left(X_{i};\theta \right)-Y_{i}{}^{GT}\right\|}_{2}^{2}$$

where θ is a set of trainable parameters in the CAFN. N is the number of the training image. $X_{i}$ is the input image and $Y_{i}$ is the ground truth density map of the image $X_{i}$. $Y\left(X_{i};\theta \right)$ means the estimated density map generated by CAFN which is parameterized with $X_{i}$. L is the loss function between estimated density map and the ground truth density map.

The network is fetched by an image of arbitrary size and outputs crowd density map. The network has two sections corresponding to the two functions, with the first part learning larger scale features and the second part restoring resolution to perform density map estimation. One of the critical components in our model is the dilated convolutional layer. Systematic dilation supports the exponential expansion of the receptive field without loss of resolution or coverage.

**Figure 3:**
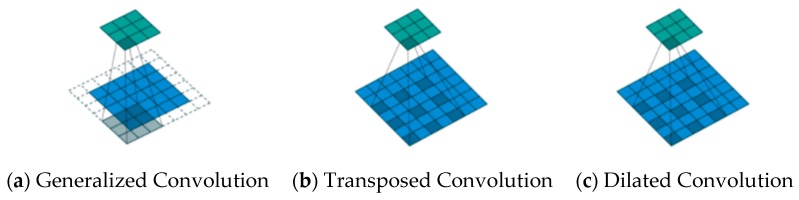
Different convolution methods [32]. Blue maps are inputs, and cyan maps are outputs. (**a**) Half padding, no strides. (**b**) No padding, no strides, transposed. (**c**) No padding, no stride, dilation.

The higher the network layer, the more information the original image contains in the unit pixel, that is, the larger the receptive field, however, which is done by pooling and takes the reduction of the resolution and the loss of the information in the original image as a cost. Due to the existence of the pooling layer, the size of the feature map in the back layer will be smaller and smaller. Dilated convolution is a kind of convolution idea that the downsampling will reduce the image resolution and the loss of information for image semantic segmentation. This feature enlarges the receptive field without increasing the number of parameters or the amount of computation. Examples can be found in Figure 3c where standard convolution (dilation rate = 1) is a 3 × 3 receptive field, and the dilated convolutions (dilation rate = 2) deliver 5 × 5 receptive fields.

The second component consists of a transposed convolution layer for up-sampling the previous layer’s output to explain the loss of details due to earlier pooling layer. Convolution arithmetic [32] is shown in Figure 3.

We apply a simple method to ensure that the improvements obtained are due to the proposed model and are not dependent on the sophisticated methods for calculating the ground truth density maps. Ground truth density map $D_{i}$ corresponding to i, the training patch is calculated by summing a 2D Gaussian kernel centered at every person’s location as defined below:

(2) $$D_{i}\left(x\right)=\sum_{x_{g}\in P}G\left(x-x_{g},\delta \right).$$

where σ is the scale parameter of the 2D Gaussian kernel and *P* is the set of all points where people are located.

### 2.3. MTCAFN Architecture

**Figure 4:**
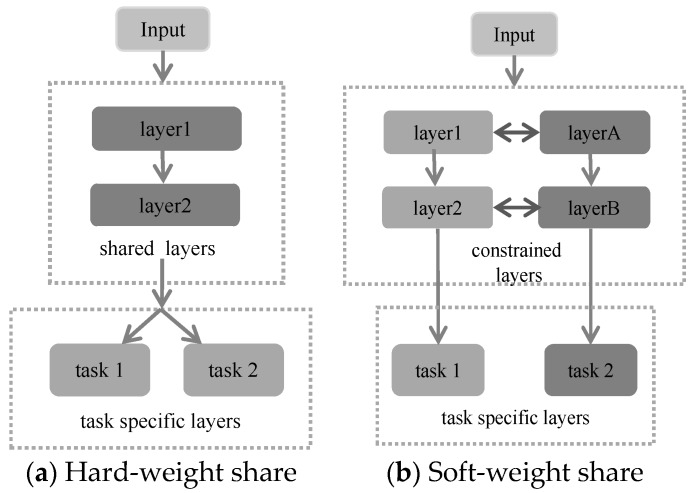
Two most used methods in multi-task learning. (**a**) Hard weight-sharing is generally applied by sharing the hidden layers between all tasks while keeping several task-specific output layers. (**b**) In soft weight-sharing, each task has its model with its parameters individually. The distance between the weights of the model is then regularized to encourage the parameters to be similar.

Since neural networks have complex network structures, training a reliable neural network requires a great deal of training data to update network parameters. However, the public datasets of crowd counting are usually included a few dozens to a few hundred samples, which results in relatively poor robustness and weak generalization of the model in some applications. Multi-task learning is a machine learning method that learns simultaneously from multiple tasks, like learning favorable information about similar tasks while effectively alleviating the problem of sparse data and improving network performance. The application of multi-task learning in deep convolution networks is mainly divided into two categories, one way is hard weight-sharing, and the alternative approach is soft weight-sharing, as seen in Figure 4. Hard weight sharing is a constraint of equal weights, while soft weight sharing means that groups of weights are encouraged to have similar values. In this paper, the hard weight-sharing is adopted to build two tasks covering crowd density estimation, as noted in Equation (3), and crowd density level classification.

Therefore a regression problem is converted into a multi-class classification problem. First, the crowd density was divided into five categories among the training data applied in this paper. Then, image classification is roughly shown as the following steps, (1) the original image is utilized as an input; (2) extracting features via the CNN; and (3) outputting the classification results by employing the Softmax classifier. The classification cross entropy is applied as the loss function in sub-network of density-level classification is demonstrated in Equation (3).

(3) $$L_{\mathrm{class}}=-\frac{1}{N}{\sum_{i=1}^{N}\sum_{j=1}^{M}T_{i,j}\log(P_{i,j})}^{},$$

where the L class represents the loss of the classification of the crowd density level, is the total number of training samples, M is the total number of categories, i is the sample, j is the category, $T_{i,j}$ is the real classification label, and $P_{i,j}$ is the prediction classification.

The crowd density and the classification loss are merged into a loss for the neural network to train and update the parameters. The function definition is shown in Equation (4):

(4) $$L=\lambda_{1}L_{\mathrm{density}}+\lambda_{2}L_{\mathrm{class}},$$

where L represents the total loss, $\lambda_{1}$ $\lambda_{2}$ are the weights of the two task loss functions respectively. Due to the difference between the loss function, the loss values obtained from the actual experiments of the two tasks are entirely different. As the crowd count is the principal task, the estimated loss of crowd density should account for a more significant proportion. Therefore, $\lambda_{1}$, $\lambda_{2}$ are used as hyper-parameters to balance the importance of two tasks.

**Figure 5:**
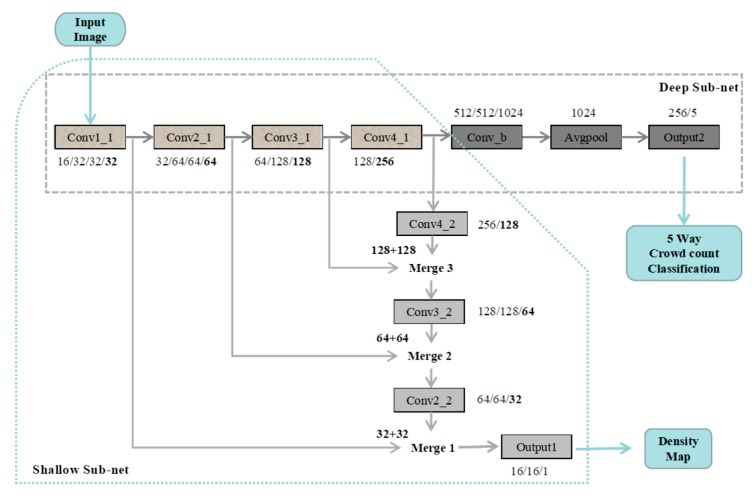
The structure of MTCAFN.

The overall structure of the MTCAFN is shown in Figure 5. It consists of a deep sub-net and a shallow one, which share convolutional layers from Conv1_1 to Conv4_1 (name of convolutional layers’ block). Grayscale images are as input to the network for reducing computational costs. The deep sub-net contains a group of convolutional layers and max-pooling to handle various sized images followed by a set of fully connected layers. The shallow sub-net includes a series of convolutional layers followed by fractional stride convolutional layers up-sampling the previous layer’s output to lower the loss of details due to earlier pooling layers. However, the loss of the shallow sub-net relies on the output of the broad network. Merge1 is an example to illustrate that Conv1_1 and Conv2_2 are merged for fusing features from different scales. In our structure, we adopted 3 × 3 (kernel size) in each layer.

Table 2 lists the layers configuration in details. The convolutional layers are denoted as “Conv2d”, specific parameters are demonstrated by the number of channels, for instance, Conv1_1 with channel 16/32/32/32, which means the layers’ block named Conv1_1 includes 4 convolutional layers connected sequentially and outputs 32 feature maps. Max-pooling layers are conducted over a 2 × 2 pixel window with stride 2. The fractional stride convolutional layer is denoted as “Upsample”, a modified linear unit ReLU is used as an activation function. The shallow network aims for crowd density estimation, while deep sub-net is for density classification. In shallow sub-net, the Conv4_2 layer block is a combination of convolutional layers, and “Upsample” applied to refine the details of the image further, while the following layer blocks in deep sub-net is with the same principal as it, which is beneficial to gain deep features thereby getting density map accurately. In deep sub-net, the Conv4_1 layer block is a combination of convolutional layers and “Max-pooling” utilized for reducing the dimension which is propitious to get abstract features so then facilitate classification, at the end the global average pooling is used to flatten the features to one dimension and output the classification results through Softmax.

## 3. Experimental Results

In this section, we propose the experimental details and evaluation results on four publicly available datasets: Shanghai Tech Part_A and Part_B [16], UCF_CC_50 [18], and WorldExpo’10 [3] dataset. For evaluation, the standard metrics used by many existing methods for crowd counting were used. MAE and MSE are applied to indicate the accuracy and robustness of estimation, respectively, where MAE is the mean absolute error and MSE is the mean squared error.

### 3.1. CAFN and MTCAFN Model Training and Testing

The proposed models are trained in a DELL PC station with a GPU Titan XP using Torch framework [33] equipped on different benchmark datasets, where variants combination to find the best way to perform the system. The Adam optimization performed during the training and evaluation with a learning rate of 0.00001 and momentum of 0.9.

**Figure 6:**
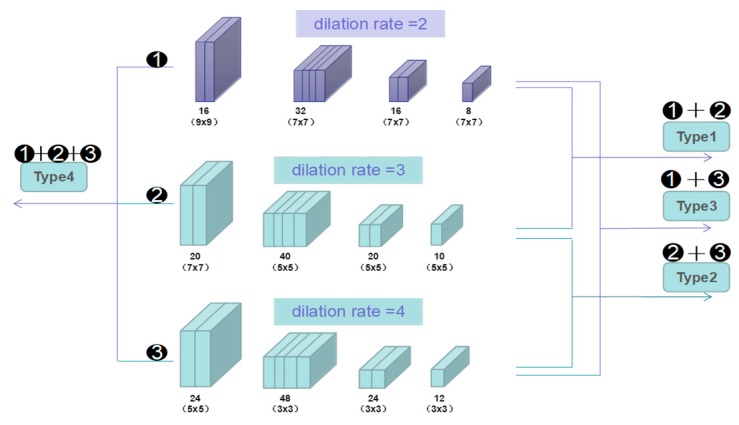
Four types of combinations.

First, four types of different column combinations (see Figure 6) with different dilate rates. Type1 is the combination of column1 (dilation rate = 2) with column2 (dilation rate = 3). Type2 is the fusion of column2 and colum3 (dilation rate = 4). Type3 combines the column1 and 3. Type4 merges all the columns. The experimental results are shown in Table 3, and we choose the Type1 model as the final way to train the CAFN network.

### 3.2. Comparison of Datasets

In Table 4, the parameters of the benchmark are listed for 3 existing datasets: Num means the number of images; Max means the maximal crowd count; Min means the minimum crowd count; Ave is the average crowd count; and Total means a total number of labeled people.

Shanghai tech dataset contains 1198 annotated images, in which a total of 330,165 people with centers of their heads interpreted. As far as we know, this dataset is the largest one regarding the number of annotated people. This dataset consists of two parts: there are 482 images in Part A, which are randomly crawled from the Internet, and 716 images in Part B, which are taken from the busy streets of metropolitan areas in Shanghai. The crowd density varies significantly between the two subsets, making an accurate estimation of the crowd more challenging than most existing datasets. Both Part A and Part B are divided into training and testing: 300 images of Part A are used for training and the remaining 182 images for testing, and 400 images of Part B are for training and 316 for testing.

Results from MTCAFN show that the double-column version achieves higher performance on Shanghai Tech Part A dataset with the lowest MAE, shown in Table 5.

The UCF_CC_50 dataset includes 50 images with different perspective and resolutions. With arriving at an average number of 1280, the number of persons annotated per image varies from 94 to 4543. Five-fold cross-validation is performed following the standard setting in Reference [16]. Result comparisons of MAE and MSE are listed in Table 6.

Zhang et al. firstly introduced WorldExpo’10 crowd counting dataset. This dataset contains 1132 annotated video sequences which are captured by 108 surveillance cameras, all from Shanghai 2010 World Expo. The author provided a total of 199,923 annotated pedestrians at the centers of their heads in 3980 frames. 3380 frames are used in training data. The testing dataset includes five different video sequences, and each video sequence contains 120 labeled frames. We train our model following the instructions are given in Section 3. Results are shown in Table 7. The proposed MTCAFN delivers the best results in Sce2 and Sce4 and promote the average MSE of 5 scenes.

### 3.3. System Verification

To validate our system on Nvidia Tx2 visualizing the experimental results, the predicted density map and the number of estimated crowd count. Compared with the ground truth and the number of labeled people. The quality of density maps is measured using two standard metrics: PSNR (Peak Signal-to-Noise Ratio) and SSIM (Structural Similarity in Image). 

**Figure 7:**
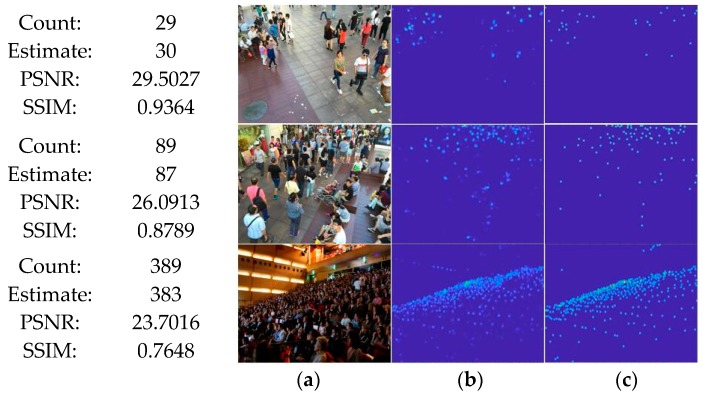
Visualization of crowd density. The corresponding test results and standard metrics are given for each row of images. The right 3 columns are described as: (**a**) Original images from Shanghai Tech dataset [16]; (**b**) Corresponding ground maps; (**c**) Estimated density maps.

The higher the two indicators are, the more representative of the generated density map is. As seen in Figure 7, due to the different shooting angles and backgrounds, the following pictures have uneven density distribution and significant scale changes. The network can still distinguish the crowd and background under harsh conditions, accurately generating density maps with quite good quality, which can prove the effectiveness of the method. Besides, our method CAFN and MTCAFN could achieve predicting results in an average of 10 frame/sec.

## 4. Conclusions

In this paper, we presented a smart camera-aware crowd counting system for jointly adopting dilated convolutions and fractional stride convolutions. Atrous convolutions are devoted to enlarging receptive fields which is beneficial to incorporate abundant characteristics into the network, which enables the model for learning globally relevant discriminative features thereby accounting for large count variations in the dataset. Additionally, we employed fractional stride convolutional layers as the back-end to restore the loss of details due to max-pooling layers in the earlier stages, therefore allowing us to regress on full resolution density maps. The model structure has moderate complexity and strong generalization ability, which possess satisfactory density estimation performance in densely crowded scenes via the experiments on multiple datasets. MTCAFN is extended where shallow sub-net extracts pixel-level detail features for crowd density map estimation, deep network extracts high-level semantic features for crowd density classification. Multi-task learning is adopted to improve the loss function of crowd counting. Through the experimental comparison, the proposed system achieved better results and verified the feasibility and effectiveness of the method.

The proposed work has some limitation as follows. A perfect alerting system focuses not only on accurate crowd counting but also on crowd behavior prediction. Moreover, local information such as sub-group trajectory analysis of the crowd also needs to be investigated. In the future, we will also focus on the compression of the broad network to fit different real-time embedding systems.

## Figures and Tables

**Table 1:** A configuration of CAFN.

Front-End (Double-Column)		Back-End
Dilation rate = 2	Dilation rate =3	No Dilation
Conv9 × 9 @ 16	Conv7 × 7 @ 20	Conv3 × 3 @ 24
Max-pooling	Max-pooling	Conv3 × 3 @ 32
Conv7 × 7 @ 32	Conv5 × 5 @ 40	ConvTranspose4 × 4@16
Max-pooling	Max-pooling	PReLU
Conv7 × 7 @ 16	Conv5 × 5 @ 20	Conv1 × 1 @ 1
Conv7 × 7 @ 8	Conv5 × 5 @ 10	Max-pooling

**Table 2:** Specific parameters of MTCAFN with Dilation Rate =2.

Layer Name	Layer Type	Channel	Output Size	Last Layer Name	Dilation
Input		1	H × W × C		
Conv1_1	Conv2d	16/32/32/32	H × W × C	Input	True, =2
Conv2_1	Maxpool + Conv2d	32/64/64/64	(H/2) × (W/2) × C	Conv1_1	True, =2
Conv3_1	Maxpool + Conv2d	64/128/128	(H/4) × (W/4) × C	Conv2_1	True, =2
Conv4_1	Maxpool + Conv2d	128/256	(H/8) × (W/8) × C	Conv3_1	True, =2
Conv4_2	Upsampling + Conv2d	256/128	(H/4) × (W/4) × C	Conv4_1	True, =2
Merge3	Concatenate	256 = (128 + 128)	(H/4) × (W/4) × C	Conv3_1, Conv4_2	
Conv3_2	Conv2d + Upsample +Conv2d	128/128/64	(H/2) × (W/2) × C	Merge3	False
Merge2	Concatenate	128 = (64 + 64)	(H/2) × (W/2) × C	Conv2_1, Conv3_2	
Conv2_2	Conv2d + Upsample +Conv2d	64/64/32	H × W × C	Merge2	False
Merge1	Concatenate	64 = (32 + 32)	H × W × C	Conv1_1, Conv2_2	
Output1	Conv2d + Maxpool +Conv2d	16/16/1	(H/2) × (W/2) × 1	Merge1	
Conv_b	Conv2d + Maxpool +Conv2d	512/512/1024	(H/8) × (W/8) × C	Conv4_1	False
Avgpool	GlobalAveragePool	1024	1024	Conv_b	
Output2	Dense + Softmax	256/5	5	Avgpool	

**Table 3:** Comparison of results on Shanghai tech dataset.

Type	Part_A		Part_B	
	MAE	MSE	MAE	MSE
Type1	100.8	152.3	21.5	38.0
Type2	103.0	161.9	24.8	45.8
Type3	99.6	155.0	28.3	48.7
Type4	101.1	160.5	24.1	45.7

**Table 4:** Parameters of the benchmark.

Num	Max	Min	Ave	Total
50	4543	94	1280	63974
3980	253	1	50	199923
482	3139	33	501	241677
716	578	9	124	88488

**Table 5:** Estimation errors on Shanghai Tech dataset.

Method	Part_A		Part_B	
	MAE	MSE	MAE	MSE
Zhang et al. [3]	181.8	277.7	32.0	49.8
Marsden et al. [34]	126.5	173.5	23.8	33.1
MCNN [16]	110.2	173.2	26.4	41.3
Cascaded-MTL [17]	101.3	152.4	20.0	31.1
Switching-CNN [22]	90.4	135.0	21.6	33.4
CAFN (ours)	100.8	152.3	21.5	33.4
MTCAFN (ours)	88.1	137.2	18.8	31.3

**Table 6:** Estimation errors on UCF_CC_50 dataset.

Method	MAE	MSE
Zhang et al. [3]	467.0	498.5
MCNN [16]	377.6	509.1
Marsden et al. [34]	338.6	424.5
Cascaded-MTL [17]	322.8	397.9
Switching-CNN [22]	318.1	439.2
CAFN (ours)	305.3	429.4
MTCAFN (ours)	303.2	417.6

**Table 7:** Estimated errors on the WorldExpo’10 dataset.

Method	Sce1	Sce2	Sce3	Sce4	Sce5	Avg.
Zhang et al. [3]	9.8	14.1	14.3	22.2	**3.7**	12.9
Shang et al. [21]	7.8	15.4	14.9	11.8	5.8	11.7
MCNN [16]	3.4	20.6	12.9	13.0	8.1	11.6
Switching-CNN [22]	4.4	15.7	10.0	11.0	5.9	9.4
CP-CNN [23]	2.9	14.7	10.5	10.4	5.8	8.9
CAFN (ours)	3.3	25.3	27.4	26.3	4.2	17.3
MTCAFN (ours)	3.4	13.8	11.2	9.7	4.8	8.2
